# Impact Ionization Properties of Polypyrrole Nanoparticles

**DOI:** 10.1021/acsearthspacechem.5c00370

**Published:** 2026-02-17

**Authors:** Rebecca Mikula, Zoltan Sternovsky, Steven P. Armes, Ethan Ayari, Derek H. H. Chan, Jordy Bouwman, Mihaly Horanyi, Sascha Kempf, Jon K. Hillier, Nozair Khawaja, Frank Postberg

**Affiliations:** † Laboratory for Atmospheric and Space Physics, 1877 University of Colorado, Boulder, Colorado 80303, United States; ‡ Smead Aerospace Engineering Sciences Department, 1877 University of Colorado, Boulder, Colorado 80303, United States; § Dainton Building, School of Mathematical and Physical Sciences, 7315 University of Sheffield, Brook Hill, Sheffield S3 7HF, U.K.; ∥ Department of Physics, 1877 University of Colorado, Boulder, Colorado 80309, United States; ⊥ Department of Chemistry, 1877 University of Colorado, Boulder, Colorado 80309, United States; # Institute of Geological Sciences, 9166 Freie Universität Berlin, 12249 Berlin, Germany

**Keywords:** polypyrrole, impact ionization, mass spectra, space, dust

## Abstract

Upcoming space missions flying dust impact ionization mass spectrometers will detect and analyze dust grains that are partially organic in composition. These organic components are expected to include mixtures of polycyclic aromatic hydrocarbons, heterocyclic compounds (containing oxygen, sulfur, and nitrogen), and additional functionalized condensed species. Dust impact ionization is a strongly velocity-dependent process that produces atomic and molecular ions reflective of the composition of the impacting particle. In this work, we characterize the impact ionization response of the nitrogen-bearing heterocyclic polymer polypyrrole (PPy). Because of its electrical conductivity, PPy is commonly used as a coating material for both mineral and organic dust particles in electrostatic dust accelerator studies. PPy nanoparticles were accelerated to velocities of 2–30 km s^–1^, and the resulting time-of-flight mass spectra were analyzed as a function of impact velocity with additional care paid to spectral variations with particle mass. The resultant mass spectra produced by impacts under roughly 8 km s^–1^ are dominated by smaller PPy-derived molecular fragments at masses 27, 28, 56, and 63u, in addition to common contaminants such as Na^+^ (23u) and K^+^ (39u). Some of these molecular fragments can be understood as originating from pyrrole, i.e., the species from which PPy is derived, while others appear to be unique to PPy. At higher velocities, the impact ionization of PPy produces two homologous series of fragment ions with the general form C_*n*_H_*m*_^+^ and C_*n*_NH_*m*_^+^, alongside the molecular fragments. This study refines our understanding of impact ionization processes for organic heterocyclic compounds and provides essential reference data for interpreting dust spectra from upcoming interstellar and interplanetary missions.

## Graphical abstract

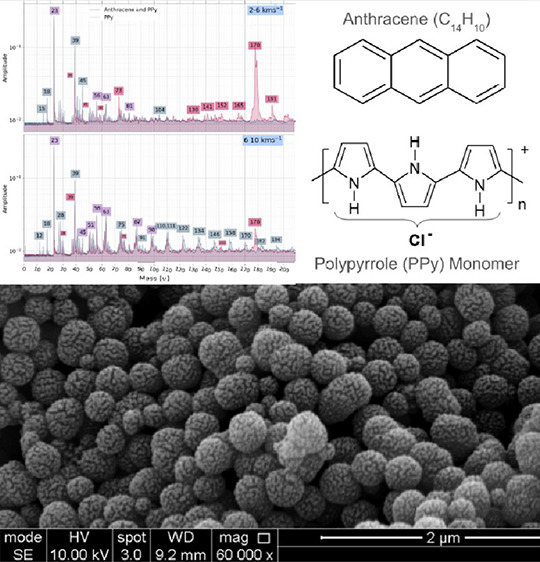

## Introduction

Three impact ionization time-of-flight mass spectrometers (the SUrface Dust Analyzer, Interstellar Dust EXperiment, and DESTINY+ Dust Analyzer) are currently either being commissioned or under development for launch into space in the near future. The science goals of these impact ionization mass spectrometers include the detection and compositional analysis of hundreds to thousands of interstellar (ISD) and interplanetary dust particles (IDPs), respectively. Such particles are expected to contain complex organic molecules, typically composed of condensed aromatic structures with functional groups (such as azoles, sulfides, and esther pthalates) and heteroatoms (e.g., N, O, or S).^1–6^ These mass spectrometers rely on impact ionization, and the ionization behavior of relevant organic compounds must be thoroughly characterized to enable accurate interpretation of data from future measurements. This study focuses on characterizing the impact ionization properties of PPy, an electrically conductive nitrogen-bearing heterocyclic polymer. In the past, PPy has been used as a coating material for dust samples to facilitate their use in electrostatic dust accelerators when the use of metallic coatings is not ideal.^7–11^ PPy is a conductive polymer that readily forms a uniform coating on both micro- and nanograins. However, in this study, it is investigated in the form of nanoparticles. The objective is to characterize the impact-ionization behavior of PPy nanoparticles as a function of impact speed to support the interpretation of data from space missions.

The Surface Dust Analyzer (SUDA) instrument was launched in 2024 onboard NASA’s Europa Clipper mission to investigate the habitability of Europa and will detect and analyze IDP particles during the cruise phase beginning in May 2027.^12^ The Interstellar Dust Experiment (IDEX) is also an impact-ionization mass spectrometer aboard NASA’s Interstellar Mapping and Acceleration Probe (IMAP) spacecraft, which launched on 24 September 2025.^13^ IMAP will operate at the Sun–Earth Lagrange point L1, conducting new observations of the inner and outer heliosphere and enabling the detection and compositional analysis of IDP and ISD particles.^14,15^ DESTINY+ (Demonstration and Experiment of Space Technology for INterplanetary voYage with Phaethon fLyby and dUst Science) is a mission currently under development that will perform a flyby of the asteroid (3200) Phaethon and analyze the dust in its vicinity.^16^ As part of the spacecraft’s payload, the DESTINY Dust Analyzer (DDA) will detect and analyze IDP and ISD particles throughout the solar system.^17^

The SUDA, IDEX and DDA instruments employ reflectron-type^18^ ion optics to analyze the composition of dust particles with high mass-resolution (*m*/Δ*m* ≥ 200).^12,13^ These instruments will detect and analyze dust particles that are partially organic in composition, most likely containing polycyclic aromatic hydrocarbons (PAHs) and related molecular species such as nitrogen-, oxygen-, or sulfur-bearing analogs.^19–22^ The presence of PAHs in the interstellar medium was first inferred from their characteristic infrared emission bands at 6.2 and 7.7 μm in the 1980s.^23,24^ More recently, a range of complex organic molecules, including PAHs and N-heterocycles have been identified in samples returned from asteroids Ryugu and Bennu.^25,26^ Similar species have been identified in Antarctic meteorite samples.^27^ In addition to various PAHs ranging from 2 to 20 fused rings, alkanes, benzene and its derivatives, carbazole, dibenzofuran, sulfides, and phthalates have been identified in meteorite and asteroidal dust samples.^28–30^ Most relevant to this study, indole and pyrrole synthesis pathways in ISM conditions have been successfully simulated in laboratory experiments.^31^ Pyrrole has long been theorized to exist in the ISM, but has not been detected via remote sensing due to a lack of significant intrinsic dipole moment.^31,32^ Pyrrole could very well act be an important component of the ISM and support the production of a variety of more complex molecules, and because of the lack of dipole moment necessary for spectroscopic observations, impact ionization mass spectrometers are well suited to searching for pyrrole and its derivatives.

The detection and compositional analysis of cosmic dust particles relies on impact ionization: a high-velocity (*v* ≥ 1 km s^–1^) impact on a solid target produces a transient ionic plasma cloud that can be measured.^33–36^ The plasma plume is chemically characteristic of both the impinging dust grain and the target material. Our understanding of the impact ionization process remains incomplete, which leads to significant uncertainties when attempting to identify the composition of impinging dust particles using their corresponding mass spectra. These uncertainties affect both inferred atomic abundances and molecular (or mineralogical) interpretations over the relevant impact velocity range (1 – 50 km s^–1^).

The impact-ionization properties of the rock-forming minerals anorthite, orthopyroxene, and olivine have been investigated previously.^37–39^ For these studies, the mineral grains were coated with a thin platinum layer to provide surface conductivity and enable acceleration in an electrostatic dust accelerator. The resulting mass spectra were recorded using laboratory versions of the instruments described above. These studies revealed distinct compositional signatures and are used to relate the measured mass spectra, including their dependence on impact velocity, to the true mineral composition. The impact ionization behavior of additional minerals coated with PPy - including iron sulfide (pyrrhotite) and aluminosilicate clay - have also been measured for the same purpose.^10,40^

Metallic overlayers cannot be easily deposited onto organic dust grains. Coating such microparticles with either platinum or palladium requires the addition of organic solvents (primarily methanol or isopropanol). The presence of such organic cosolvents can cause the in situ dissolution of many organic dust grains.^41^ Organic microparticles are instead coated with organic conductive polymers, such as PPy. The organic species polystyrene, polyanaline, poly­(4-bromostyrene), poly­(methyl methacrylate), anthracene, and PPy have been the subject of impact ionization mass spectrometry experiments.^7,9–11,40,42^ Each of the referenced studies concludes a lack of pyrrole ring features in the mass spectra. There is disagreement whether PPy coatings contribute to the mass spectra. Typically, studies focusing on organic core particles concluded that PPy makes no significant contribution.^9,11,40,42^ Hillier et al.,^10^ however, conclude that when used as a coating, PPy does significantly contribute to the resultant mass spectra. This difference may be attributed to the relative ease of disentangling organic from mineral species, as compared to distinguishing between organic–organic species. The present study is particularly relevant to a recent investigation by Mikula et al.,^11^ which examined the impact ionization characteristics of PPy-coated anthracene microparticles (Figure 1).^43^

**Figure 1:**
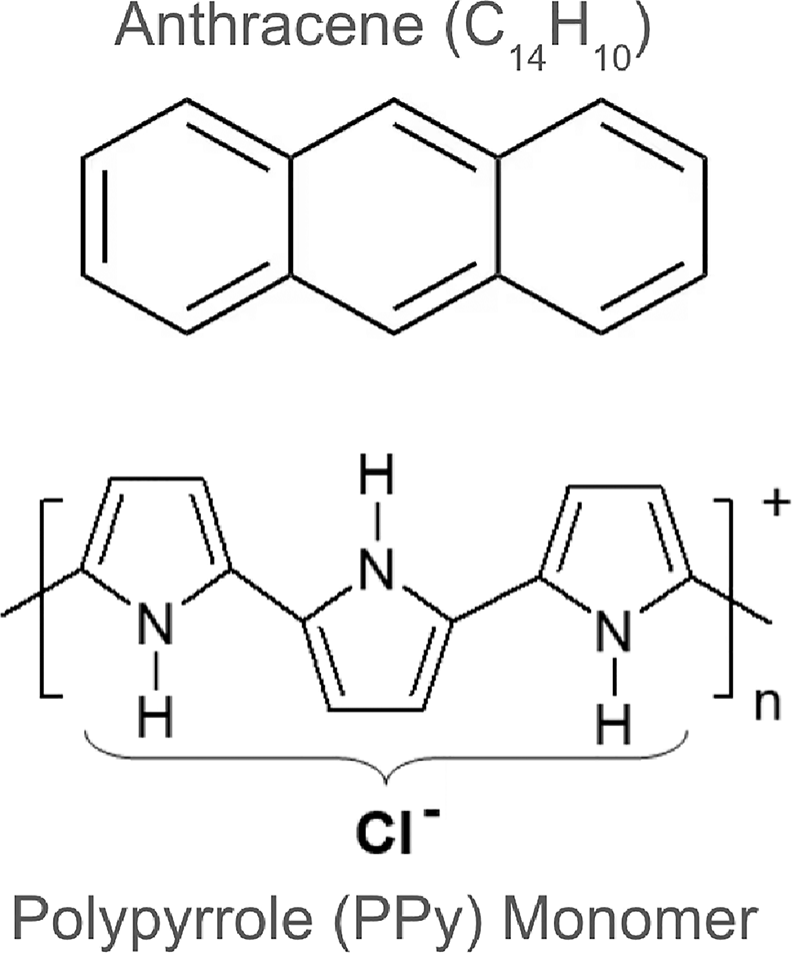
Chemical structures of anthracene and polypyrrole (PPy). It is worth noting that although both species contain aromatic rings, PPy lacks the fused-ring framework characteristic of anthracene and other PAHs.

Mikula et al. demonstrated that anthracene can be reliably identified by impact ionization mass spectrometry over the velocity range of 2–8 km s^–1^.^11^ Under such conditions, characteristic mass lines corresponding to the anthracene radical cation, C_14_H_10_^+^, and protonated cation, C_14_H_11_^+^, were observed at 178 and 179u, respectively. Mass lines at 191 and 203u were assigned to the clusters (C_14_H_10_· CH)^+^ and ((C_14_H_10_· CCH)^+^ respectively. The final identifying feature was the mass line at 152u, which is consistent with the well-known PAH fragmentation C_2_H_2_ loss pathway to form either biphenylene or its isomer, cyclobuta­[b]­naphthalene. These features constrain the mass and number of rings present in the parent PAH molecule. It is also likely that some of the features attributed to anthracene can instead be more directly explained by the fragmentation of the PPy coating.

Molecular fragmentation increases as a function of impact velocity. Large quantities of hydrocarbons and elemental carbon and hydrogen are produced on impact as neutrals, cations, and anions. In principle, these small fragments and atomic species may collide within the plasma to generate the observed homologous series of molecular ions. It is also feasible that such homologous series originate from random bond cleavages within the PPy coating.^7,10^ The random cleavage of C–C bonds between pyrrole rings (plus C–N bonds within pyrrole rings) combined with cluster chemistry within the plasma provides a reasonable explanation for the homologous series observed in the impact ionization mass spectra.

The organic nature of the PPy coating suggests a number of important questions that were beyond the scope of Mikula et al. In particular, it was not clear whether the observed homologous series was characteristic of anthracene (and perhaps other PAHs) or whether it represented characteristic impact ionization behavior for organic molecules more generally. Moreover, the relationship between the impact ionization spectral features of organic molecules and the original mass of the impinging particles has not yet been systematically investigated. Traditionally, impact ionization mass spectral features are considered to depend only on the grain’s initial impact velocity.^35^

Additional studies investigating the impact ionization of PPy both as a coating material and as a nanoparticle include the work of Hillier et al. and Srama et al.^10,44^ Both studies concluded that molecular fragments produced by the dissociation of PPy can dominate the mass spectra of PPy-coated microparticles. In particular, Hillier et al. proposed that mass lines in cationic spectra at 27, 28, 56, 57, 58, 66, 73, 81, 93, and 105u can be attributed, at least in part, to PPy dissociation. The results from the present study further support and extend these earlier findings on the impact ionization mass spectra of PPy nanoparticles and PPy-coated microparticles.

In this study, we characterize the intrinsic impact ionization behavior of the PPy coating material by performing dedicated measurements on PPy nanoparticles. These findings are then used to examine the interplay between the PPy and anthracene components previously reported by Mikula et al. More specifically, we investigate the velocity dependence of selected mass features and their contribution to the total ion content. We also identify species that are direct products of impact ionization of PPy rather than of anthracene (or a mixture of anthracene and PPy).

While dust impact ionization is distinct in its strong velocity dependence and the characteristic fragmentation and/or clustering patterns of the resulting ions, it is helpful to compare these findings with previous fragmentation and dissociation studies of pyrrole (C_4_H_5_N, mass 67u). Mass spectra obtained via electron impact ionization,^46^ photoionization,^45^ and pyrolysis^47^ are considered for comparison. The five prevailing mass lines produced via electron impact ionization, in decreasing abundance, are the parent ion at mass 67u C_4_H_5_NH^+^, followed by mass 39u (C_2_NH^+^), 41u (C_2_NH_3_^+^), 40u (C_3_H_4_^+^ and C_2_NH_2_^+^), and 28u (CNH_2_^+^). The photoabsorption and photodissociation of pyrrole, combined with ToF mass spectrometry of the resulting ions, was studied by Rennie et al. over a photon energy range of 11.8–27.5 eV.^45^ The major ion species are identical to those observed in electron impact ionization, but their relative abundances depend on photon energy (Figure 2). Pyrrole has an ionization threshold of approximately 8.2 eV, and the parent ion dominates the ToF spectrum at photon energies below about 12.5 eV. Fragment ions become the dominant products at energies above roughly 13.5 eV. The pyrolysis of pyrrole was studied over a temperature range of 1260–1710 K in a mixture with Ar gas, and the major products identified were H_2_, C_2_H_2_ (26u), HCN (27u), C_3_H_4_ (40u), and C_2_H_3_N (41u). In summary, these studies reveal the pyrrole fragmentation pathways and that they are robust across various mechanisms.

**Figure 2:**
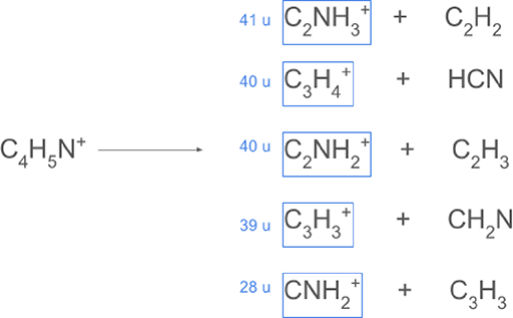
Characteristic dissociation pathways for pyrrole, which is the heterocyclic monomer repeat unit for polypyrrole (PPy), as reported by Rennie et al.^45,46^ The cationic fragments are seen in photolysis and electron ionization and dissociation mass spectra. These species likely contribute to the impact ionization mass spectra, see text for detail.

## Measurements and Analysis

### Polypyrrole (PPy) Dust Sample

The PPy dust sample was prepared following the protocol reported by Cawdry et al.^48^ 1.0 g of poly­(ethylene oxide) (PEO; molecular weight 4 × 10^6^g mol^–1^ was dissolved in water (90 mL) with the aid of magnetic stirring. Separately, FeCl_3_·6H_2_0 was dissolved in 10 mL of deionized water and added to the aqueous PEO solution, yielding an orange-brown mixture. Pyrrole monomer (1.0 mL) was then added and allowed to polymerize at 20 °C for 12 h.

The resulting black dispersion was purified by three centrifugation–redispersion cycles (10,000 rpm, 20 min each) to remove excess PEO, Fe­(II) salts, and unreacted pyrrole. The purified aqueous dispersion was then freeze-dried overnight, yielding a fine black powder. The resulting particles contained more than 95% PPy by mass (with the remainder being PEO). A representative SEM image of these PEO-stabilized PPy particles is shown in Figure 3.

**Figure 3:**
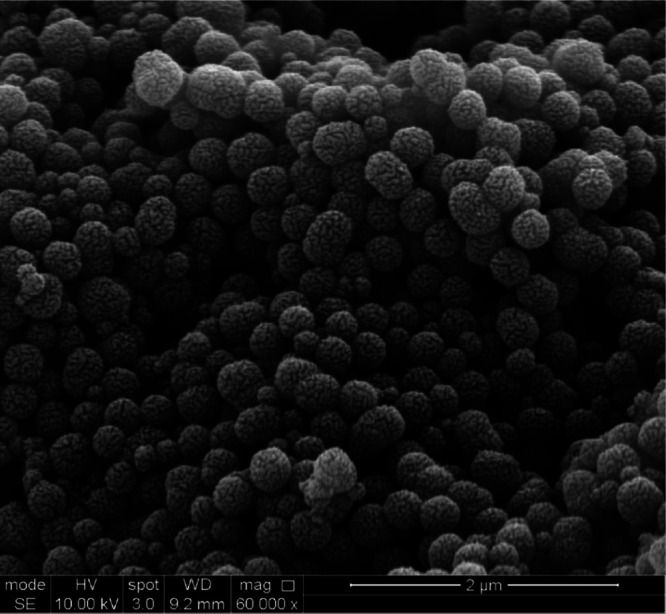
Scanning electron microscope (SEM) image of the PPy dust sample. The image shows submicron-sized particles, which are capable of reaching high velocities in the dust accelerator. The distinctive surface texture is an artifact owing to the thin layer of sputtered gold applied to the sample to prevent charging effects during SEM analysis.

### Dust Accelerator Measurements

No unexpected or unusually high safety hazards were encountered during the experimental dust campaign to collect mass spectra for this study. The impact ionization mass spectra of the PPy particles were recorded using the same experimental setup as that described for the PPy-coated anthracene particles in Mikula et al. Briefly, a laboratory prototype of the IDEX instrument was used for the measurements, with impact ionization occurring on a high-purity gold-coated target surface. The IDEX prototype is nominally operated in cationic mode with a mass resolution upward of 150 at mass 200 u. The PPy particles were accelerated to velocities ranging from 2 to 30 km s^–1^, with a mass vs velocity distribution shown in Figure 4. The characteristic of this distribution reflects the behavior of the dust accelerator, in which higher velocities can be reached only for particles of lower mass.^49^ The facility provides the velocity and mass for each particle, and particle size (radius) is calculated assuming a PPy density of ρ = 1.45 g cm^–3^, as determined by helium pycnometry.^43^

**Figure 4:**
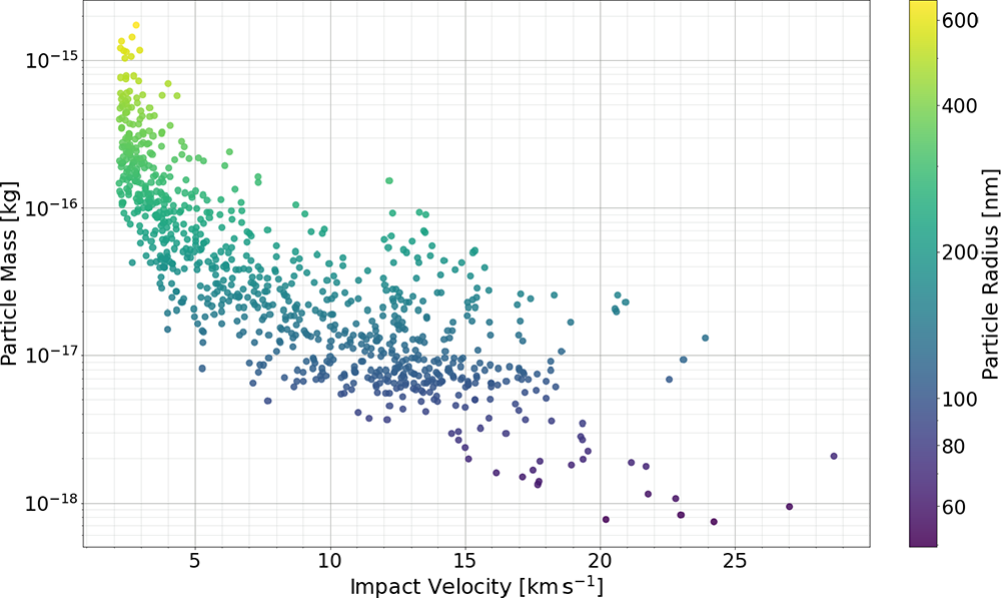
Mass–velocity distribution obtained for all the particles analyzed in this study. This data set is then reduced to 276 particles according to certain criteria. See text for details.

From a total of 965 ToF mass spectra, 276 were selected for further analysis. Selection criteria included the absence of plasma artifacts (e.g., bulges or smearing in the raw ToF signal), reliable particle mass and velocity measurements, and the presence of multiple mass lines with signal-to-noise ratios (SNRs) greater than 3. The spectra were analyzed using an analytical protocol similar to that described by Mikula et al. For each analyzed particle, a summary report was generated containing its mass, size, and impact velocity, along with a list all identified of mass lines. For each identified mass line, the associated mass and fractional ion contribution relative to the total ToF signal are recorded.

## Results

Co-added spectra within narrow velocity ranges provide a useful overview of impact ionization features and their variation with impact velocity, as shown in Figures 5 and 6. Each co-added spectrum was constructed from 30 individual spectra within the appropriate velocity bin, except for the two highest-velocity bins, which were constructed using 15 spectra each. Mass spectra binned in 2 km s^–1^ velocity increments are self-similar for spectra produced by velocities less than 20 km s^–1^; co-addition eliminates minor variations between individual spectra while enhancing features that are characteristic of the overall ionization behavior. Spectra produced by impacts with velocities greater than 20 km s^–1^ can be binned in 10 km s^–1^ increments.

**Figure 5:**
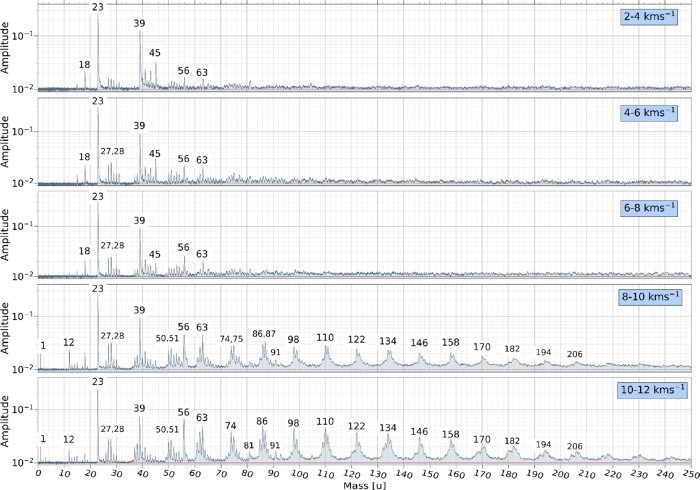
Co-added impact ionization mass spectra recorded for PPy particles within narrow velocity bins between 2 and 12 km s^–1^ with major signals labeled in atomic mass units.

**Figure 6:**
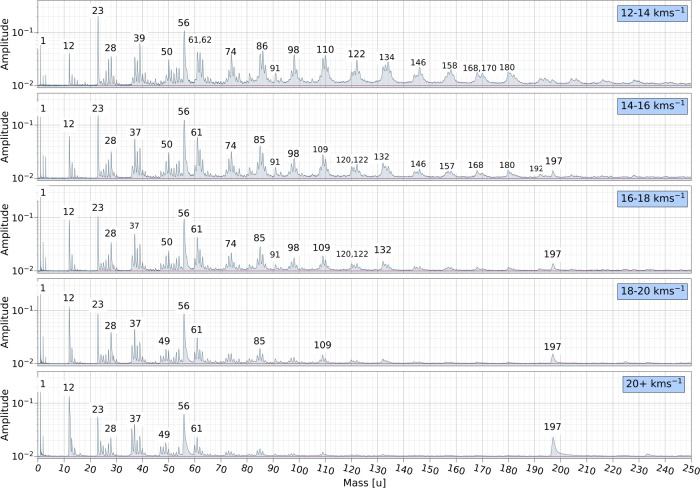
Co-added impact ionization mass spectra recorded for PPy particles within narrow velocity bins between 12 and 20 + km s^–1^ with major signals labeled in atomic mass units.

The gradual appearance and disappearance of distinct features observed in the mass spectra shown in Figures 5 and 6 is characteristic of the impact ionization behavior of organic materials. At lower velocities, the kinetic energy of the impinging particles is insufficient to break all the chemical bonds within the (macro)­molecules. In this case, only partial bond cleavage occurs and weaker bonds are more likely to be broken than stronger bonds. For example, PPy-coated polystyrene microparticles traveling at 5 km s^–1^ undergo cleavage of C–C and C–H bonds in the polystyrene backbone and the PPy coating. Pendant phenyl groups in polystyrene tend to form stable aromatic species such as the tropylium cation. This sort of fragmentation increases with increasing impact velocity until kinetic energies are sufficient to dissociate the majority of both C–C bonds between rings within the polymers as well as aromatic bonds within the rings. This occurs around 18 km s^–1^ as fragmentation becomes more complete, and the corresponding mass spectra tend to be dominated by atomic mass lines and mass lines associated with a few highly stable fragments.^7,9,40^ This effect becomes more pronounced with increasing velocity above 18 km s^–1^. These prior observations inform the present study.

In contrast to the observations made by Mikula et al. for PPy-coated anthracene microparticles, the cation mass spectra recorded for the PPy nanoparticles shown in Figure 5 do not contain any “parent cations”—representing either a PPy monomer, PPy polymer of arbitrary length *n*, or pyrrole ring—at impact velocities below 8 km s^–1^. Instead, the latter spectra are dominated by signals assigned to surface contaminants on both the target and the impacting particles, including alkali metal cations such as Na^+^ (23u) and K^+^ (39u). An additional mass line at 18u can most likely be attributed to ammonium (NH_4_^+^) with a very minor contribution from water (H_2_O^+^) adsorbed to the target surface. The weakening of the mass line at mass 18u as a function of increasing impact velocity combined with the lack of mass lines at 16, 17, and 19u (representing O^+^, OH^+^, H_3_O^+^, respectively) indicates that the bulk of ions associated with the 18u line are produced by ammonium rather than water. Additionally, we do not expect the presence of mass lines associated with NH_3_^+^, NH_2_^+^, NH^+^, N^+^. N–H bonds are significantly weaker than O–H bonds, so while we see O–H species we do not expect analogous N–H species to appear. Additionally, at high impact velocities, we assign masses 14 and 15 u to CH_2_^+^ and CH_3_^+^ respectively, There is not sufficient mass resolution to resolve potential peaks associated with CH_2_^+^ and N^+^ or CH_3_^+^ and NH^+^ respectively. In addition, nitrogen has a higher ionization energy than oxygen. While O^+^ has been observed at impact velocities greater than approximately 15–20 km s^–1^ N^+^ would not be possible until impact velocities reached 25–30 km s^–1^.^39^

As noted above, the PPy-coated anthracene microparticle and PPy nanoparticle spectra are qualitatively different at low impact velocities (*v* < 8 km s^–1^). Anthracene reliably produces a parent cation (*M* ^+^) plus related hydrogenated species ([*M* + 1]^+^) under such conditions. The mass lines originating from PPy in this velocity range correspond to fragmentation products observed at *m*/*z* 27, 28, 56, and 63u. These peaks are attributed to the species listed in Table 1. C_2_H_3_^+^ is a relatively stable vinyl cation is readily produced via PPy dissociation. While C_2_H_3_^+^ could also result from anthracene dissociation, mass 27u is not particularly enhanced in mass spectra produced by PPy-coated anthracene microparticles when compared to PPy nanoparticles. This is discussed in more detail in the Discussion section Polypyrrole Coating Effects. The 28u peak is also observed in the photoionization and pyrolysis mass spectra recorded for pyrrole.^45,47^ On the other hand, the 56u mass line is attributed to C_*n*_N_*x*_H_*m*_^+^ species and iron, given the low probability of forming C_4_H_8_^+^ via dissociation of PPy or clustering. Formation of a species representative of C_4_H_8_^+^ would require a pyrrole ring to be isolated, stripped of nitrogen, and heavily hydrogenated. Finally, the 63u signal could be attributed to either C_5_H_3_^+^ or C_4_NH^+^.C_4_NH^+^ is much more likely as a fragmentation product of PPy than C_5_H_3_^+^, which would require a multistep mechanism to produce. C_5_H_3_^+^ could, however form via clustering.

**Table 1:** Possible Ion Fragments Produced from PPy Impacts in the Velocity Range 2–8 km s^–1^

mass [u]	possible C_*n*_H_*m*_^+^ species	possible C_*n*_N_*x*_H_m_^+^ species
27	C_2_H_3_^+^ (likely)	CNH^+^
28	C_2_H_4_^+^ (likely)	N_2_^+^ or CNH_2_^+^
56	C_4_H_8_^+^	C_3_NH_6_^+^ (likely) or C_2_N_2_H_4_^+^
63	C_5_H_3_^+^	C_4_NH^+^ (likely)

At impact velocities below 10 km s^–1^, there are three important conclusions that can be drawn.

1. The mass spectra are largely dominated by features attributed to Na^+^ and ^39^K^+^. Masses 39 and 41u never reproduce ion ratios consistent with potassium isotopes. It then follows that mass lines at 39, 40, and 41u are a mixture of K^+^, Ca^+^, and the PPy dissociation products C_3_H_3_^+^, C_3_H_4_^+^, C_2_NH_2_^+^, and C_2_NH_3_^+^.
2. Relatively high amplitudes for the 56u line is attributed to C_3_NH_6_^+^ and iron. The organic fragment is unique to the impact ionization dissociation of PPy and is not observed in mass spectra associated with pyrrole dissociation via other mechanisms.
3. PPy does not produce a characteristic parent cation due to its polymeric nature. Additionally, there is no evidence of pyrrole ring formation or intact PPy chains of arbitrary length n.

Similar exercises can be performed for mass lines commonly observed at impact speeds of 8–16 and 16–30 km s^–1^. Over the velocity range 8–16 km s^–1^, mass lines observed at 23, 39, 40, 41, 56, 61, 62, 63 are consistent with those described in the text above and Table 1. Mass lines at masses ranging from 73 to 192u appear and become more intense with increasing velocity. The majority of these lines (those at 74, 86, 98, 110u and so on up to 206u) are part of the homologous series of C_*n*_H_*m*_^+^ and C_*n*_NH_*m*_^+^ fragments and clusters. Additional mass lines with relatively lower amplitudes appear at 81, 91, and 93u. These mass lines can be attributed to PPy fragmentation in which a single heterocycle remains intact and subsequent clustering of such fragments with the free ions C^+^, CH^+^, and CH_3_^+^. The associated free ions can be produced via PPy dissociation or via dissociation of organic contaminants adsorbed to the target surface. Likely chemical compositions of these clusters are C_5_H_7_N^+^, C_6_H_5_N^+^, and C_6_H_7_N^+^ respectively. Finally, a line at mass 197u appears and gradually increases in amplitude. This line is entirely attributed to Au^+^ from the target.

The final velocities to consider range over 16–30 km s^–1^. The features that contribute most to the total ion content are H^+^, C^+^, Na^+^, Au^+^, plus mass lines at 36, 37, 56, and 61u. Signals 36 and 37u can be attributed to C_3_^+^ and C_3_H^+^ while mass 61u is most likely attributed to C_5_H^+^. At this point, the homologous series has more or less disappeared. Instead, a few relatively stable atomic species dominate via contributions from H^+^, C^+^, and so forth. It is noteworthy that the mass line at 56 amu remains a remarkably stable feature across the measured conditions. However, at these impact velocities, the feature is primarily attributed to iron rather than organic molecular fragments. The iron in this case can be entirely attributed to the FeCl_3_·6H_2_O used in the production of the nanoparticles. These results are supported Goldsworthy et al. and Hillier et al.^10,40^ The vast majority of ion content in impact ionization spectra produced by high speed impacts are elemental and have little fragment contribution. The most prominent species in each study are H^+^, C^+^, Na^+^, the target material, and mass lines at 39 and 56 u.

## Discussion

### Polypyrrole Coating Effects

Polypyrrole is one of the most commonly used coating materials dust samples in electrostatic dust accelerator studies.^10,40,50^ With characteristic PPy impact ionization features now established, the contribution to spectra from a PPy coating can be more accurately assessed. Mass spectra from PPy-coated anthracene microparticles reported by Mikula et al. were obtained under the same experimental conditions used for the PPy nanoparticle spectra shown above.^11^ Hillier et al. similarly detected and analyzed PPy nanoparticles and PPy-coated aluminosilicate clay microparticles using a reflectron dust impact ionization mass spectrometer.^10^ Together, these data sets enable an assessment of how PPy coatings influences resultant mass spectra.

Co-added mass spectra originating from both PPy-coated anthracene microparticles and PPy nanoparticles were overlaid according to four velocity bins: 2–6, 6–10, 10–16, and 16 + km s^–1^ (Figures 7 and 8). PPy-coated anthracene spectra are plotted in red, PPy nanoparticle spectra are shown in blue, and lines that overlap with high correspondence appear as purple. Labels for mass lines are colored accordingly. Impacts at low velocities *v* < 16 km s^–1^ were normalized to the Na^+^ line at 23u, while higher speed impacts were normalized to H^+^ at mass 1u.

**Figure 7:**
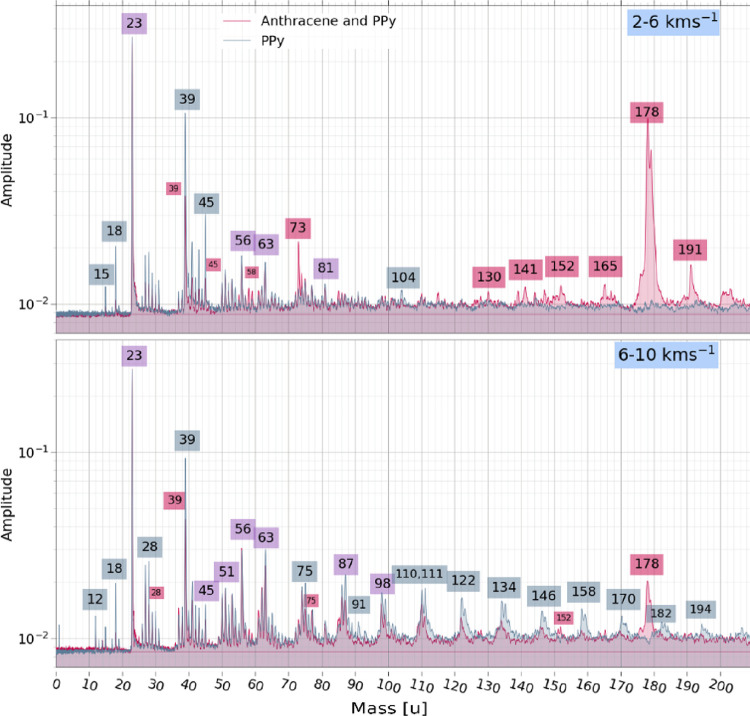
Co-added spectra recorded for PPy nanoparticles (blue) and PPy-coated anthracene microparticles (red) overlaid for the following impact velocity ranges: 2–6 and 6–10 km s^–1^ (from top to bottom). Features labeled in purple are observed at the same relative intensities in both PPy nanoparticle and PPy-coated anthracene microparticle spectra. See main text for further details.

**Figure 8:**
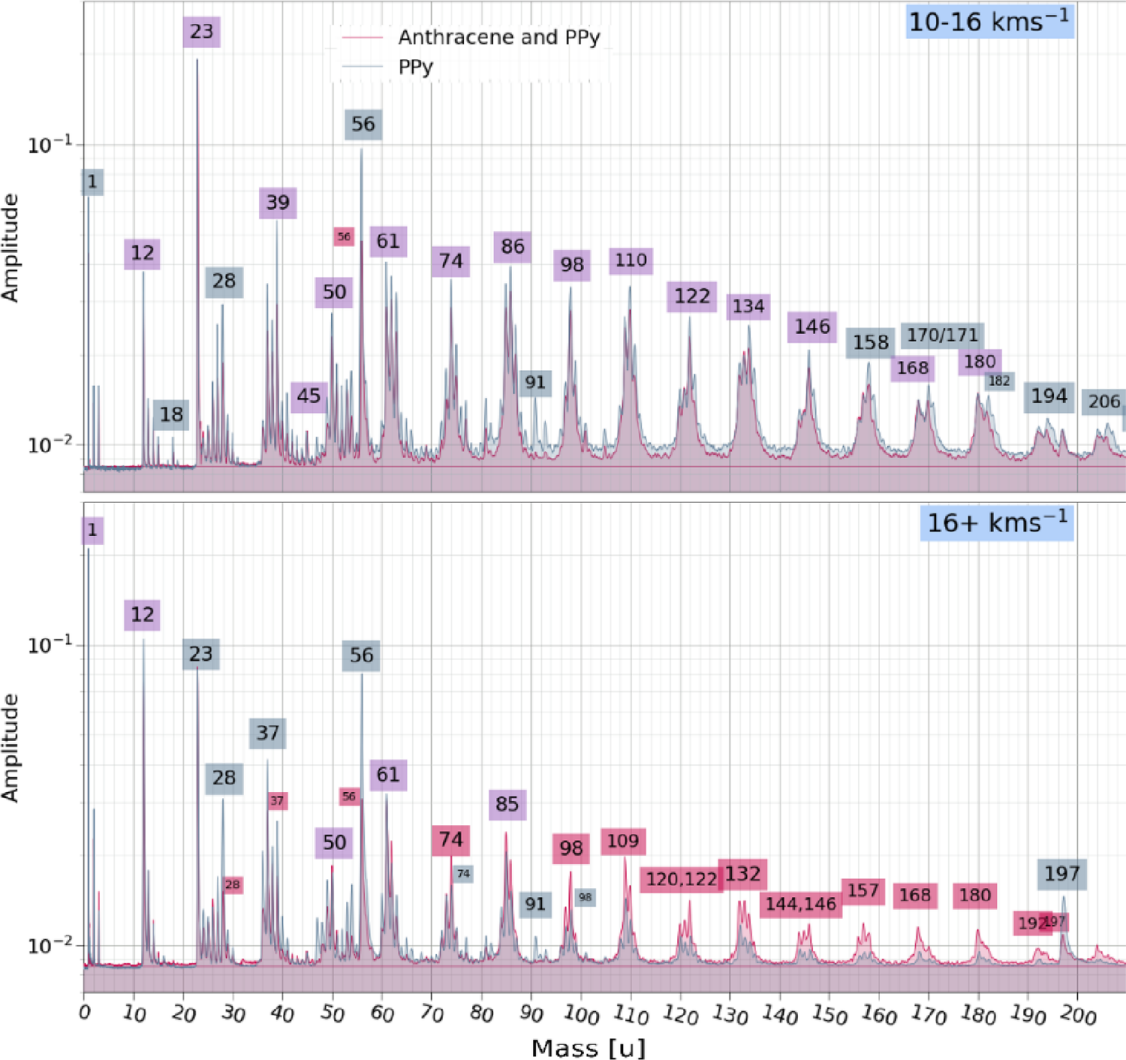
Co-added spectra recorded for PPy nanoparticles (blue) and PPy-coated anthracene microparticles (red) overlaid for the following impact velocity ranges: 10–16, and 16 + km s^–1^ (from top to bottom). Features labeled in purple are observed at the same relative intensities in both PPy nanoparticle and PPy-coated anthracene microparticle spectra. See main text for further details.

Spectra produced from impacts in the range 2–6 km s^–1^ are the most distinctive. A high amplitude feature spanning 178–179u is shown in red. This represents the anthracene parent cation (or its isomer) and the parent mass plus one mass unit. Anthracene fragments consistent with known PAH dissociation pathways are also observed at masses 130, 141, 152, and 165u. The last set of features are anthracene-derived CH clusters starting at 191u. We also see a weak feature at mass 104u (shown in blue). Blue labels have been added to masses 15, 18, 39, and 45u to indicate that these features have significantly higher amplitudes in the PPy nanoparticle spectra compared to those observed in spectra recorded for the PPy-coated anthracene microparticles. Finally, species that are roughly 1:1 in terms of amplitude and total ion content in spectra obtained for the PPy-coated anthracene microparticles or the PPy nanoparticles are shown in purple. Such features include mass lines at 56, 63, and 81u.

Accordingly, we can begin to disentangle the origins of most of the mass lines present (Table 2). Mass lines that most likely originate from PPy (rather than anthracene or target contamination) include 56 and 81u. It also becomes clear that masses 39, 40, 41, and 45u are enhanced in the PPy nanoparticle spectra compared to the anthracene microparticles. The most pronounced enhancement is seen at mass 39u. This is likely due to increased C_3_H_3_^+^; a major dissociation product seen in pyrolysis, photodissociation, and electron impact dissociation of pyrrole.^45–47^ In principle, C_3_H_3_^+^ can be generated from anthracene dissociation, but it is much more likely to be produced by PPy. Anthracene primarily dissociates via C_2_H_2_ loss to form either biphenylene or cyclobuta­[b]­naphthalene radical cations.^51^ The secondary loss pathway (C_4_H_2_ loss) forms a naphthalene radical cation. C_2_H_2_ and C_4_H_2_ could undergo collisions in the plasma plume to form C_3_H_3_^+^, but this species is not representative of a direct anthracene fragment.

**Table 2:** Summary of Mass Lines Observed in Co-Added Impact Ionization Mass Spectra Recorded for Both PPy-Coated Anthracene Microparticles and PPy Nanoparticles at Impact Velocities of 2–8 km s^–1^

mass [u]	probable species	probable source
15	CH_3_^+^, NH^+^	target, anthracene, PPy
18	H_2_O^+^, NH_4_^+^	target, PPy
23	Na^+^	target
39	^39^K^+^, C_3_H_3_^+^, C_2_NH^+^	target, PPy
45	C_2_H_4_OH^+^, C_2_NH_7_^+^	target, PPym, and production contaminants
56	C_3_NH_6_^+^, Fe^+^	PPy and production contaminants
63	C_5_H_3_^+^	anthracene, PPy
130	C_10_H_10_^+^	anthracene
141	C_11_H_9_^+^	anthracene
152	C_12_H_8_^+^	anthracene
165	C_13_H_9_^+^	anthracene
178	C_14_H_10_^+^	anthracene
191	[C_14_H_10_· CH]^+^	anthracene

Impact ionization spectra produced by particles impacting at velocities upward of ∼8 km s^–1^ do not produce the characteristic anthracene parent ion, clusters, or fragments. Instead, we observe a homologous series of C_*n*_H_*m*_^+^ peaks. These species were originally attributed almost exclusively to anthracene in Mikula et al. It is now clear that such features cannot be solely attributed to anthracene. Figures 7 and 8 show that both PPy-coated anthracene microparticles and PPy nanoparticles produce the same homologous series reported in Mikula et al. It is certainly feasible that C_*n*_H_*m*_^+^ clusters are produced by anthracene (and perhaps other PAHs) but we demonstrate herein that this feature is reproduced in PPy nanoparticle mass spectra. Furthermore, it is quite possible that the series is instead produced via random dissociation within PPy.

Given that PPy nanoparticles produce a clear C_*n*_H_*m*_^+^ series, it is necessary to consider how PPy coatings contribute to the total mass fraction of PPy-coated microparticles. In Mikula et al., the anthracene particles were coated in a PPy layer with a mean thickness of approximately 20 nm. As a result, smaller particles inherently contain a higher mass fraction of PPy; for example, particles smaller than 100 nm consist of at least 50*%* PPy by mass. Among the PPy-coated anthracene microparticles, very few particles traveling faster than 8 km s^–1^ remain primarily anthracene by mass (Figure 9). In addition, some of the apparently small particles may represent detached PPy shell fragments generated during surface charging prior to acceleration.

**Figure 9:**
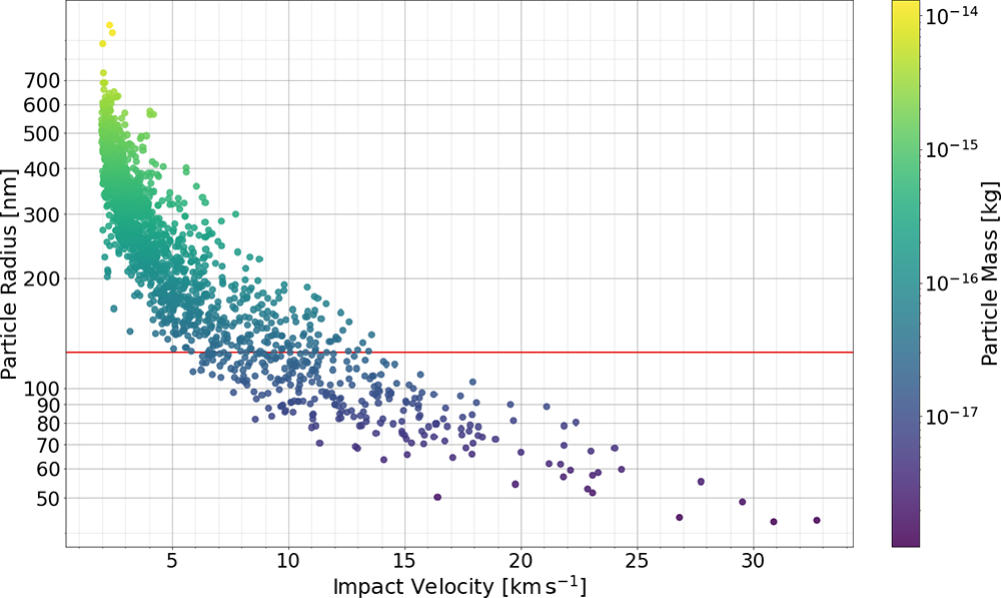
Mass vs velocity distribution of 1667 particles recorded for use in Mikula et al. The red line represents the approximate mass at which particles become ≈50*%* PPy by mass.

In view of these complications, mass–velocity profiles such as that shown in Figure 9, should be used in conjunction with mean particle sizes estimated via electron microscopy. This provide a practical means of defining a realistic lower particle mass threshold. Such a threshold helps distinguish genuine PPy-coated microparticles with core–shell morphology from PPy-rich nanoparticles or detached PPy shell fragments.

### Relationship between Impactor Mass and Impact Chemistry

Mass lines resulting from impact ionization are not solely dependent on particle velocity, particle mass also plays a significant role. This is because collisions are more likely to occur within denser impact ion plumes, which are associated with correspondingly more massive particles. For example, two PPy nanoparticles with masses of 5.2 and 384 fg respectively moving at 9.9 km s^–1^ can produce mass spectra with qualitatively different features (Figure 10).

**Figure 10:**
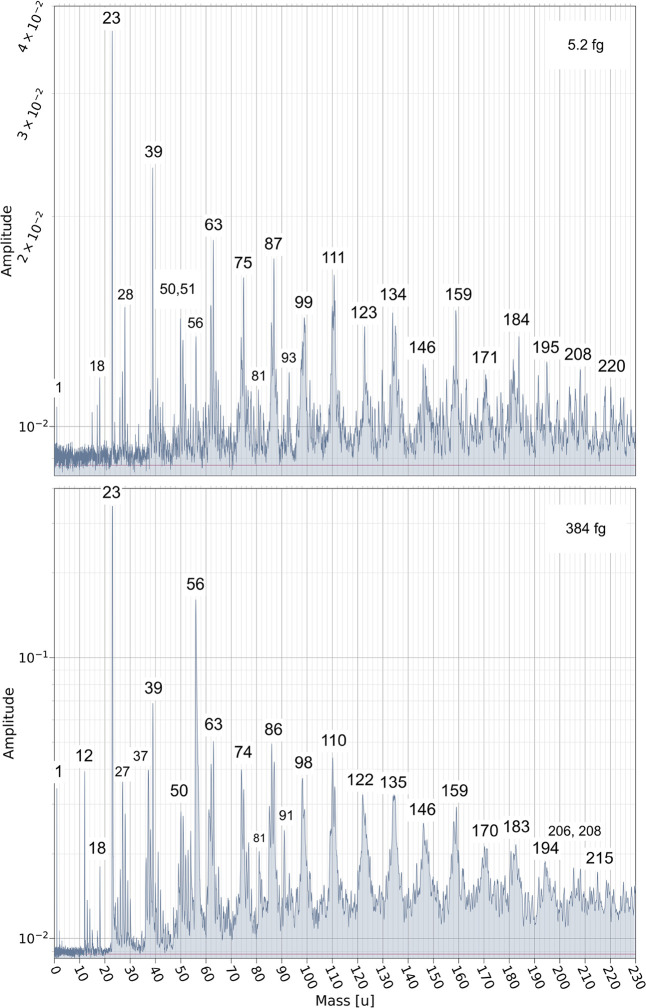
Individual spectra recorded at 9.9 km s^–1^ for PPy nanoparticle masses of 5.2 and 384 fg, respectively. Note that the spectrum associated with the 384 fg particle generally exhibits significantly higher amplitudes. However, there is proportionally more ion content associated with features at mass 56u and the homologous series peaks slightly shift in mass by ±1 u.

The most prominent evidence for such mass dependence is the enhanced series pattern observed in spectra produced by more massive particles within a narrow velocity range. This is revealed by coadding spectra produced by PPy nanoparticles with velocities of 9–10 km s^–1^ that were further binned according to particle size (Figure 11).

**Figure 11:**
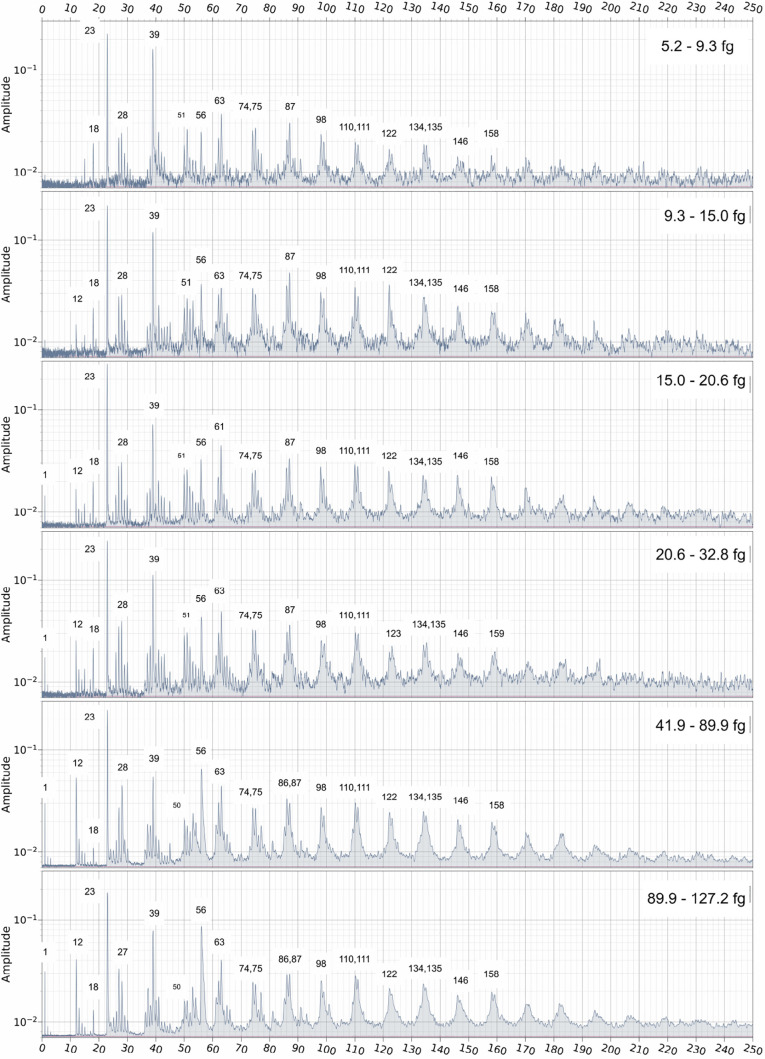
Co-added mass spectra produced by PPy nanoparticles of varying masses/radii within a velocity range of 9–10 km s^–1^.

From the classical point of view, all impact ionization spectra should be effectively identical (apart from random variations plus plasma features such as lower mass resolution and bulge effects for large particles.^34,35^ Instead, enhanced homologous series, shifting series maximum peaks (i.e 86 vs 87), and disproportionately higher amplitude features associated with ion cloud chemistry are observed (for example, see the mass lines at 56, 77, 81, 91, and 93u). The C_7_^+^ series is the most noticeable example of peaks shifting for larger particles. At the low end of the particle size range (≈100 nm) the major signal in the C_7_^+^ series is located at 87u. This corresponds to either C_7_H_3_^+^ or C_6_NH^+^. However, this spectral feature is gradually replaced by a mass line at 86u for more massive particles, which represents either C_7_H_2_^+^ or C_6_N^+^ species.

Identifying which species are responsible for the changes in amplitude/ion content is beyond the scope of the current study. It is important to note that these changes occur, as this is the first convincing evidence for the effect of particle mass on impact ionization spectra. Additionally, we must make clear that impact velocity is the dominant factor in impact ionization, but it now becomes evident that dust grain mass plays a larger role than previously thought.

### The Presence of Trace Contaminants Versus PPy Dissociation Fragments

We also note the presence of species that can be attributed to solvents, oxidizing agents, and other materials used in the production of the bulk PPy nanoparticle powder. These species are treated as contaminants with regard to the PPy, as the goal was to produce high-purity PPy nanoparticles and any additional material within the grains constitutes an unwanted impurity. From this perspective, the most prominently seen contaminant is iron. The iron in these spectra comes directly from the FeCl_3_·6H_2_0 used in particle synthesis. Although iron represents a very minor contribution in terms of total mass, it is readily observed in the spectra, particularly at higher impact velocities. (Figure 12).

**Figure 12:**
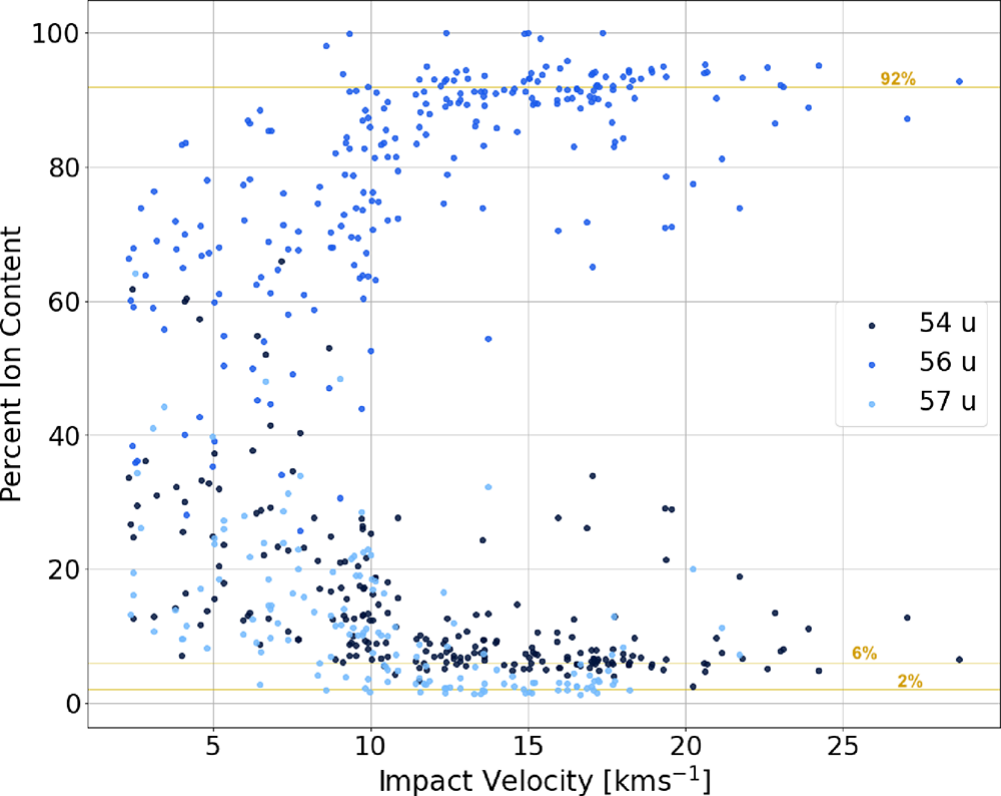
We added the ion contents associated with masses 54, 56, and 57 u. From this figure, we can see the progression of the ratio of these species as a function of impact velocity. At impact velocities greater than roughly 12 km s^–1^, we see that the ion content ratios approach those of terrestrial iron.

We cannot assume the entirety of the mass 56 u signals comes from iron. First, iron does not consistently appear in impact-ionization mass spectra until impact velocities reach at least 10.6 km s^–1^.^38^ Below this velocity threshold, iron is observed only sporadically in the spectra, even when iron is a relatively major contributor. Second, PPy (and other organic molecules) can produce fragments or clusters with masses of 54, 56, or 57 u. These fragments likely contribute significantly to the relevant mass lines at impact velocities below 12 km s^–1^. The lack of consistent iron ionization alone is not enough to explain the discrepancies in ion ratios between standard terrestrial iron and what we record.

## Conclusions

Polypyrrole has been used for more than two decades as a conductive coating for use in dust impact ionization mass spectrometry studies. It is also an excellent mimic for nitrogen-bearing heterocycles in cosmic dust samples. The primary results from this study can be summarized in four points.

1. Impact ionization of PPy nanoparticles produces a distinct line at mass 56u that is attributed to C_3_NH_6_^+^. This feature is likely to be diagnostic of nitrogen-bearing heterocyclic compounds.
2. At impact velocities less than 8 km s^–1^ PPy nanoparticles produce features that are consistent with those observed for electron impact ionization dissociation and photodissociation with the addition of the novel fragmentation products C_3_NH_6_^+^ and C_5_H_3_^+^ at masses 56 and 63u, respectively. The addition of strong features at masses 56 and 63u indicate a dissociative mechanism distinct to impact ionization.
3. Whether present as nanoparticles or as a coating material, PPy produces a distinctive homologous series of C_*n*_H_*m*_^+^ (and/or C_*n*_NH_*m*_^+^) species ranging from *n* = 2 up to at least *n* = 16 at impact velocities in the range 8–18 km s^–1^. In principle, this series of hydrocarbon clusters could be generated either within the plasma cloud or via random bond cleavage of C–C and C–N bonds within the polymer chains. Thus, for impact velocities greater than 8 km s^–1^ PPy cannot be distinguished from the core organic aromatic species when used as a coating for PAH molecules such as anthracene.
4. In addition to its impact velocity, the mass of the initial PPy nanoparticle also influences the resulting impact ionization spectra.

The unambiguous identification of pyrrole and its derivatives is a more difficult task for dust mass analyzers due to the lack of a “parent ion” and subsequent clusters. Unfortunately, potentially diagnostic species such as CN^–^ and CN rich species are located in the anionic component of the plasma, which is both beyond the scope of this study and not applicable to the IDEX instrument. The most useful constraining cationic feature observed in PPy impact ionization mass spectra seems to be the prominent mass line at 56u, which is consistent with iron isotopic ratios only for high velocity impacts (*v* > 12 km s^–1^). The cluster nominally associated with C_4_^+^ warrants further investigation because it might be a “fingerprint” that is characteristic of pyrrole rings.

When using PPy as a coating material, due care should be taken to prevent the incorrect assignment of PPy features to the core material. For example, when PPy is used as a coating material for anthracene, mass lines at 56 and 63u may primarily originate from PPy rather than the PAH. This does not mean that any spectral features observed at both 56 and 63u are solely attributable to PPy but rather that appropriate caution must be taken when analyzing mass spectra from inhomogeneous dust grains. The same logic must be followed when dealing with organic *C*_*n*_ *H*_*m*_^+^ homologous series. Although the homologous series shown in both this study and Mikula et al. can be derived from random PPy dissociation, the formation equivalent clusters from PPy, anthracene, other PAHs, or even other organic compounds cannot be ruled out for masses below roughly 100u. This supports the similar conclusion made by Hillier et al. It is worthwhile to note that this work and Hiller et al. directly contradict the Goldsworthy et al. and Burchell et al. conclusions that PPy overlayers do not contribute significantly to the resultant mass spectrum.^9,40^ This discrepancy may perhaps be explained by the fact that Burchell et al. considers only particles impacting at less than 8 km s^–1^ for in depth analysis. The contributions from PPy to the overall mass spectra are only pronounced above roughly 10 km s^–1^. Goldsworthy et al. included results from PPy-coated particles with impact velocities greater than 10 km s^–1^. However, due to the polymeric nature of PPy, we can infer that PPy and other polymeric species, such as polystyrene and polyaniline, produce similar homologous series arising from either clustering or C–C bond cleavage. These origin of the homologous series cannot be distinguished between polymers.

Finally, the presence of the C_*n*_H_*m*_^+^ series is dependent not only on the incident velocity but also on the mass of the impinging PPy nanoparticle. Larger particles of higher mass invariably produce mass features with higher amplitudes within a given velocity range. This is presumably due to a higher plasma density being generated immediately after impact. New experimental campaigns focused on a wider range of PAHs (e.g., pyrene and perylene), alternative conductive polymers (e.g., polyaniline), and other assorted organics (e.g., substituted carbazoles) should be beneficial in determining whether these phenomena also hold for a wider range of organic materials.

## References

[1] Sandford S. A., Engrand C., Rotundi A. (2016). Organic matter in cosmic dust. Elements.

[2] Flynn G., Keller L., Feser M., Wirick S., Jacobsen C. (2003). The origin
of organic matter in the solar system: Evidence from the interplanetary
dust particles. Geochim. Cosmochim. Acta.

[3] Flynn G., Keller L., Wirick S., Jacobsen C. (2008). Organic matter in interplanetary
dust particles. Proceedings of the International
Astronomical Union.

[4] Matrajt G., Messenger S., Brownlee D., Joswiak D. (2012). Diverse forms
of primordial
organic matter identified in interplanetary dust particles. Meteoritics & Planetary Science.

[5] Mann I. (2010). Interstellar
dust in the solar system. Annual Review of Astronomy
and Astrophysics.

[6] Guélin M., Cernicharo J. (2022). Organic molecules
in interstellar space: Latest advances. Frontiers
in Astronomy and Space Sciences.

[7] Burchell M. J., Willis M., Armes S. P., Khan M. A., Percy M., Perruchot C. (2002). Impact ionization experiments with
low density conducting
polymer-based micro-projectiles as analogues of solar system dusts. Planetary and Space Science.

[8] Goldsworthy B., Burchell M. J., Cole M. J., Green S. F., Leese M., McBride N., McDonnell J., Müller M., Grün E., Srama R. (2002). Laboratory
calibration
of the cassini cosmic dust analyser (CDA) using new, low density projectiles. Adv. Space Res..

[9] Burchell M. J., Armes S. P. (2011). Impact ionisation spectra from hypervelocity
impacts
using aliphatic poly (methyl methacrylate) microparticle projectiles. Rapid Commun. Mass Spectrom..

[10] Hillier J. K., Sternovsky Z., Armes S. P., Fielding L. A., Postberg F., Bugiel S., Drake K., Srama R., Kearsley A. T., Trieloff M. (2014). Impact ionisation
mass spectrometry of polypyrrole-coated
pyrrhotite microparticles. Planetary and Space
Science.

[11] Mikula R., Sternovsky Z., Armes S. P., Ayari E., Bouwman J., Chan D. H. H., Fontanese J., Horanyi M., Hillier J. K., Kempf S., Khawaja N., Kupihár Z., Postberg F., Srama R. (2024). Impact ionization mass spectra of
polypyrrole-coated anthracene microparticles: a useful mimic for cosmic
polycyclic aromatic hydrocarbon dust. ACS Earth
Space Chem..

[12] Kempf S. (2025). SUDA: A SUrface Dust
Analyser for Compositional Mapping of the Galilean
Moon Europa. Space Sci. Rev..

[13] Horányi M., Tucker S., Sternovsky Z., Tyagi K., Knappmiller S., Ayari E., Mikula R., Szalay J. R., Kempf S., Bollendonk C., Gurnee R., Fisher M., McCann D., Coakley L., Gurst S., Davis K., Bramer S., Miller M., Neher J., Newcomb G., Oberg S., Sayler J., Wade S., Deaton T., Doner A., Fontanese J., Grün E., Miller C., Wing R., Holguin L., Fowle M., Hellickson T., Rhode A., Brennan N., Hansen D., O’Connor D., Kostovny A., Shaver S., Looney K., Turner N. J., Hillier J., Postberg F., Srama R., Christian E., Gkioulidou M., McComas D. J., Schwadron N. A. (2025). Interstellar Dust Experiment (IDEX) Onboard NASA’s Interstellar
Mapping And Acceleration Probe (IMAP). Space
Sci. Rev..

[14] McComas D., Christian E. R., Schwadron N. A., Fox N., Westlake J., Allegrini F., Baker D., Biesecker D., Bzowski M., Clark G. (2018). Interstellar mapping
and acceleration probe (IMAP): A new NASA mission. Space Sci. Rev..

[15] McComas D. J. (2025). Interstellar Mapping
And Acceleration Probe: The NASA IMAP Mission. Space Sci. Rev..

[16] Ozaki N., Yamamoto T., Gonzalez-Franquesa F., Gutierrez-Ramon R., Pushparaj N., Chikazawa T., Tos D. A. D., Çelik O., Marmo N., Kawakatsu Y., Arai T., Nishiyama K., Takashima T. (2022). Mission design of DESTINY+: Toward active asteroid
(3200) Phaethon and multiple small bodies. Acta
Astronaut..

[17] Simolka J., Blanco R., Ingerl S., Krüger H., Sommer M., Srama R., Strack H., Wagner C., Arai T., Bauer M. (2024). The DESTINY+
Dust Analyser—a
dust telescope for analysing cosmic dust dynamics and composition. Philos. Trans. R. Soc., A.

[18] Mamyrin B., Karataev V., Shmikk D., Zagulin V. (1973). The mass-reflectron,
a new nonmagnetic time-of-flight mass spectrometer with high resolution. Zh. Eksp. Teor. Fiz..

[19] Allamandola L., Tielens A., Barker J. (1989). Interstellar polycyclic aromatic
hydrocarbons—The infrared emission bands, the excitation/emission
mechanism, and the astrophysical implications. Astrophys. J. Suppl. Ser..

[20] Thomas K. L., Keller L. P., Blanford G. E., McKay D. S. (1994). Quantitative analyses
of carbon in anhydrous and hydrated interplanetary dust particles. AIP Conf. Proc..

[21] Chiar J., Tielens A., Adamson A., Ricca A. (2013). The structure, origin,
and evolution of interstellar hydrocarbon grains. Astrophysical Journal.

[22] McGuire B. A., Burkhardt A. M., Kalenskii S., Shingledecker C. N., Remijan A. J., Herbst E., McCarthy M. C. (2018). Detection of the
aromatic molecule benzonitrile (c-C6H5CN) in the interstellar medium. Science.

[23] Allamandola L., Tielens A., Barker J. (1985). Polycyclic aromatic hydrocarbons
and the unidentified infrared emission bands-Auto exhaust along the
Milky Way. Astrophys. J..

[24] Allamandola L., Sandford S., Wopenka B. (1987). Interstellar polycyclic aromatic
hydrocarbons and carbon in interplanetary dust particles and meteorites. Science.

[25] Zeichner S. S., Aponte J. C., Bhattacharjee S., Dong G., Hofmann A. E., Dworkin J. P., Glavin D. P., Elsila J. E., Graham H. V., Naraoka H. (2023). Polycyclic
aromatic hydrocarbons in samples of Ryugu
formed in the interstellar medium. Science.

[26] Glavin D. P., Dworkin J. P., Alexander C. M. O., Aponte J. C., Baczynski A. A., Barnes J. J., Bechtel H. A., Berger E. L., Burton A. S., Caselli P. (2025). Abundant
ammonia and nitrogen-rich soluble organic
matter in samples from asteroid (101955) Bennu. Nature Astronomy.

[27] Becker L., Glavin D. P., Bada J. L. (1997). Polycyclic aromatic
hydrocarbons
(PAHs) in Antarctic Martian meteorites, carbonaceous chondrites, and
polar ice. Geochim. Cosmochim. Acta.

[28] Aponte, J. ; Buckner, D. ; Mojarro, A. ; Elsila, J. ; Glavin, D. ; Dworkin, J. ; CConnolly, Jr H. ; Lauretta, D. Two-Dimensional Gas Chromatography Reveals Complex Organic Astrochemistry in A Bennu Sample. In 56th Lunar and Planetary Science Conference, 2025.

[29] Danger G., Ruf A., Maillard J., Hertzog J., Vinogradoff V., Schmitt-Kopplin P., Afonso C., Carrasco N., Schmitz-Afonso I., D’hendecourt L. L. S. (2020). Unprecedented molecular
diversity revealed in meteoritic insoluble organic matter: the Paris
Meteorite’s case. Planetary Science Journal.

[30] Sephton M. A., Chan Q. H., Watson J. S., Burchell M. J., Spathis V., Grady M. M., Verchovsky A. B., Abernethy F. A., Franchi I. A. (2024). Insoluble macromolecular organic
matter in the Winchcombe
meteorite. Meteoritics & Planetary Science.

[31] Wang J., Nikolayev A. A., Marks J. H., Turner A. M., Chandra S., Kleimeier N. F., Young L. A., Mebel A. M., Kaiser R. I. (2024). Interstellar
Formation of Nitrogen Heteroaromatics [Indole, C8H7N; Pyrrole, C4H5N;
Aniline, C6H5NH2]: Key Precursors to Amino Acids and Nucleobases. J. Am. Chem. Soc..

[32] Kutner M. L., Machnik D. E., Tucker K. D., Dickman R. L. (1980). Search for interstellar
pyrrole and furan. Astrophys. J..

[33] Drapatz S., Michel K. W. (1974). Theory of Shock-Wave Ionization upon High-Velocity
Impact of Micrometeorites. Zeitschrift f ü
r Naturforschung A.

[34] Hornung K., Kissel J. (1994). On shock wave impact ionization of dust particles. Astron. Astrophys..

[35] Auer A., Sitte K. (1968). Detection technique for micrometeoroids using impact ionization. Earth and Planetary Science Letters.

[36] Auer, S. In Interplanetary Dust; Grün, E. , Gustafson, B. Å. S. , Dermott, S. , Fechtig, H. , Eds.; Springer: Berlin, 2001; pp 385–444.

[37] Hillier J., Postberg F., Sestak S., Srama R., Kempf S., Trieloff M., Sternovsky Z., Green S. (2012). Impact ionization mass
spectra of anorthite cosmic dust analogue particles. J. Geophys. Res.: Planets.

[38] Fiege K., Trieloff M., Hillier J. K., Guglielmino M., Postberg F., Srama R., Kempf S., Blum J. (2014). Calibration
of relative sensitivity factors for impact ionization detectors with
high-velocity silicate microparticles. Icarus.

[39] Hillier J. K., Sternovsky Z., Kempf S., Trieloff M., Guglielmino M., Postberg F., Price M. C. (2018). Impact ionisation mass spectrometry
of platinum-coated olivine and magnesite-dominated cosmic dust analogues. Planetary and Space Science.

[40] Goldsworthy B., Burchell M. J., Cole M. J., Armes S. P., Khan M. A., Lascelles S., Green S. F., McDonnell J., Srama R., Bigger S. (2003). Time of flight
mass spectra of ions
in plasmas produced by hypervelocity impacts of organic and mineralogical
microparticles on a cosmic dust analyser. Astronomy
& Astrophysics.

[41] Hillier J. K., Sestak S., Green S., Postberg F., Srama R., Trieloff M. (2009). The production of platinum-coated
silicate nanoparticle
aggregates for use in hypervelocity impact experiments. Planetary and Space Science.

[42] Burchell M. J., Cole M. J., Lascelles S. F., Khan M. A., Barthet C., Wilson S. A., Cairns D. B., Armes S. P. (1999). Acceleration of
conducting polymer-coated latex particles as projectiles in hypervelocity
impact experiments. J. Phys. D: Appl. Phys..

[43] Chan D. H., Millet A., Fisher C. R., Price M. C., Burchell M. J., Armes S. P. (2021). Synthesis and characterization of
polypyrrole-coated
anthracene microparticles: a new synthetic mimic for polyaromatic
hydrocarbon-based cosmic dust. ACS Appl. Mater.
Interfaces.

[44] Srama R., Woiwode W., Postberg F., Armes S. P., Fujii S., Dupin D., Ormond-Prout J., Sternovsky Z., Kempf S., Moragas-Klostermeyer G. (2009). Mass spectrometry
of hyper-velocity impacts of organic micrograins. Rapid Communications in Mass Spectrometry: An International Journal
Devoted to the Rapid Dissemination of Up-to-the-Minute Research in
Mass Spectrometry.

[45] Rennie E., Johnson C., Parker J. E., Ferguson R., Holland D., Shaw D. (1999). A photoabsorption and mass spectrometry study of pyrrole. Chem. Phys..

[46] NIST, Pyrrole National Institute of Standards and Technology Chemistry WebBook, SRD 69. https://webbook.nist.gov/cgi/cbook.cgi?ID=C109977Mask=200Mass-Spec.

[47] Hong X., Zhang L., Zhang T., Qi F. (2009). An experimental and
theoretical study of pyrrole pyrolysis with tunable synchrotron VUV
photoionization and molecular-beam mass spectrometry. J. Phys. Chem. A.

[48] Cawdry, N. ; Obey, T. M. ; Vincent, B. Colloidal dispersions of electrically conducting polypyrrole particles in various media J. Chem. Soc., Chem. Commun. 1988, 1189. 10.1039/C39880001189

[49] Shu A., Collette A., Drake K., Grün E., Horányi M., Kempf S., Mocker A., Munsat T., Northway P., Srama R. (2012). 3 MV hypervelocity
dust
accelerator at the Colorado Center for Lunar Dust and Atmospheric
Studies. Rev. Sci. Instrum..

[50] Fielding L. A., Hillier J. K., Burchell M. J., Armes S. P. (2015). Space science applications
for conducting polymer particles: synthetic mimics for cosmic dust
and micrometeorites. Chem. Commun..

[51] West B., Sit A., Mohamed S., Joblin C., Blanchet V., Bodi A., Mayer P. M. (2014). Dissociation
of the anthracene radical cation: a comparative
look at iPEPICO and collision-induced dissociation mass spectrometry
results. J. Phys. Chem. A.

